# Power Load Probabilistic Prediction Based on Multi-Value Quantile Regression and Timing Fusion Ensemble Learning Model

**DOI:** 10.3390/e28030329

**Published:** 2026-03-16

**Authors:** Yuhang Liu, Fei Mei, Jun Zhang, Xiang Dai, Wen Li

**Affiliations:** School of Electrical and Power Engineering, Hohai University, Nanjing 211100, China; 231306080028@hhu.edu.cn (Y.L.); 231606030078@hhu.edu.cn (J.Z.); 241606010057@hhu.edu.cn (X.D.); 241306020063@hhu.edu.cn (W.L.)

**Keywords:** load forecasting, probabilistic forecasting, temporal fusion model, multi-value quantile regression, ensemble learning

## Abstract

The core component to ensure the refined and safe operation of distribution network scheduling is 10 kV bus load probabilistic prediction. However, existing probabilistic prediction methods suffer from insufficient dynamic feature extraction and compromised prediction reliability caused by quantile crossing. To address these issues, this paper proposes a 10 kV bus load probabilistic prediction method integrating multi-value quantile regression (MQR) and a temporal fusion ensemble learning model (ELM). Firstly, a temporal fusion ensemble learning model is constructed, which integrates multiple temporal fusion network (TFN) sub-models through a stacking framework to parallel extract multi-dimensional temporal features of loads, effectively enhancing its feature capture capability for complex load data. Secondly, MQR is introduced as the core objective function to synchronously generate multi-quantile load forecasting results, comprehensively depicting the load probability distribution. Finally, a Listwise Maximum Likelihood Estimation (ListMLE) ranking constraint mechanism is embedded, which optimizes quantile ordering through monotonicity constraints, significantly reducing the degree of quantile crossing and improving the interpretability of forecasting results. The results show that the MQR-ELM algorithm achieves a Prediction Interval Coverage Probability of 94.624% (close to the nominal coverage rate of 95%), a Prediction Interval Averaged Width of 588.526, a Crossing Degree Index of only 0.0476, and a Continuous Ranked Probability Score as low as 84.931. All core indicators are significantly superior to those of the comparative algorithms.

## 1. Introduction

With the continuous advancement of the construction of China’s new power system, the safe operation of the distribution network is of great significance to national security, social stability and economic development [1,2]. The 10 kV busbar is an important part of the distribution network. Achieving accurate prediction of the 10 kV busbar load is a key link in the stable operation and economic benefits of the distribution network. Unlike system load, the base value of the 10 kV busbar load is small, with large fluctuations, and is influenced by various factors. In recent years, the large-scale penetration of distributed power sources such as renewable energy into the power grid has significantly increased the uncertainty of the 10 kV busbar load, which intensifies the complexity of information uncertainty in load data. Traditional deterministic load prediction [3] has certain limitations when describing load uncertainty. Load probabilistic forecasting, as a typical application of information theory in engineering practice, aims to quantify the randomness and volatility of loads by analyzing the uncertainty of load-related information, and is also an important technical support for improving the flexibility and reliability of distribution networks [4].

Furthermore, against the backdrop of electricity market reform and competitive regulatory pressures, probabilistic load forecasting demonstrates great practical application value in downstream decision-making to address market uncertainties. High-precision prediction intervals and continuous probability density estimations not only serve the physical security of the power distribution network but also provide market participants, such as power retailers, power generation enterprises, and demand-side response aggregators, with quantified risk boundaries. In modern power markets, this refined uncertainty information directly provides solid data support for strategic scheduling, unit commitment, and day-ahead bidding frameworks. As the power system transitions to a more complex environment, the uncertainty of forecasting fundamentally interacts with the complex network evolution dynamics that drive technological diffusion and strategic adaptation among power enterprises [5]. Based on high-precision probabilistic load forecasting, a deep learning model integrated with game theory can effectively analyze the strategic interactions and dynamic adaptation processes among electricity purchasing and selling enterprises [6], optimize the electricity purchasing and selling behaviors of market entities, and finally achieve economically optimal decision-making in the energy market. For load probabilistic prediction, research can be carried out considering the two aspects of model and method. The methods include probability parameter estimation [7], interval prediction [8,9], and quantile regression [10,11]. Parameter estimation mainly focuses on the statistical characteristics of model parameters, but the information that it provides for load probabilistic prediction is not sufficient. Interval prediction can directly predict upper and lower limit intervals, and its prediction results can provide limits for load fluctuations, but it struggles to provide information on the probability density of the load and the probability distribution. To obtain more load uncertainty information, predicted values and the probability density [12] at different quantile levels can be obtained through quantile regression and kernel density estimation, thereby providing the confidence interval and probability distribution of the load. The model includes a parametric model [13], a data-driven model [14], and a non-parametric model [15]. The probabilistic prediction method, which is based on the parametric model, uses an assumption of the load distribution to predict parameters, and can directly obtain the probability density or cumulative distribution curve of the load based on the model’s output parameters [16]. However, the parametric model relies very much on the rationality of the distribution assumption. In real-world situations, it is unlikely that the load at a certain moment will strictly obey a certain distribution. Data-driven models focus on directly learning load fluctuation changes from data. There is no need to assume data distribution in advance. Their performance depends on data quality and sample size. In Ref. [17], a power load forecasting model named BilineAR-DRTFT is proposed. By introducing a variety of demand response signals and other new engineering features, as well as an improved Bilinear feedforward network, the researchers effectively improve the prediction accuracy of the model. However, in the absence of an adequate data volume, the performance of data-driven models will significantly decrease, and such characteristics are usually more complicated [18]. Non-parametric models do not require a hypothetical distribution of loads, have relatively low requirements for data, have extensive applicability, and have received widespread attention from scholars in China and abroad in recent years. This model is generally built using quantile regression combined with deep learning algorithms. Ref. [19] proposed a load probabilistic prediction method based on quantile regression forest and quantile determination, which can generate multiple-quantile prediction results in parallel, solving the reliability problem of direct prediction intervals when building prediction intervals. To improve the prediction deviation of quantile regression in extremely small and extremely large quantile values, Ref. [20] combined parametric models with non-parametric models to effectively solve this type of problem.

Currently, state-of-the-art deep learning architectures, such as Transformer variants, temporal convolutional networks (TCNs), and hybrid attention-recurrent models, have been widely applied in time-series forecasting. However, these architectures exhibit certain limitations when dealing with 10 kV bus loads. While Transformer models excel at capturing long-term dependencies, they are computationally intensive and often overlook local temporal anomalies. TCNs effectively extract local patterns but lack flexibility when processing multivariate heterogeneous inputs, such as combining meteorological factors with historical loads. In contrast, the TFN was selected as the core feature extractor in this study because it is specifically designed for multivariate, multi-horizon time-series forecasting. Its variable selection network can adaptively filter out redundant noise from meteorological data, while the integration of LSTM and multi-head attention mechanisms allows it to simultaneously balance short-term local load fluctuations and long-term periodic dependencies. These architectural characteristics are highly consistent with the severe volatility and strong external-factor susceptibility of 10 kV bus loads.

Quantile regression in most studies currently provides only predicted values under a certain quantile and cannot fully describe the entire probability distribution of load. Therefore, the concept of multi-value quantile regression was proposed. Multi-value quantile regression can predict load prediction results corresponding to future load quantiles and more comprehensively describe the probability distribution of a load. For different quantile load prediction values, theoretically higher quantiles correspond to higher load prediction values, but due to quantile crossing [21], a low quantile prediction value may be higher than a high quantile prediction value, thereby weakening the rationality of the prediction result. Existing research reduces the crossover by the re-arrangement of quantile values or by adding sorting constraints to the loss. In addition, since the multi-value quantile regression model includes multi-objective tasks such as prediction of different quantile values and sorting constraints, a single model cannot accurately capture all dynamic features, and the prediction performance of the model would have large deviations with different initialization values. The integrated learning model can effectively solve this problem. The integrated learning model better adapts to different data distributions and features by training multiple sub-models, and integrates the advantages of multiple sub-models to reduce deviation and variance in a single model, improving the accuracy and generalizability of predictions. At present, the integrated learning model has achieved good results in point prediction models, including the Boosting model based on decision tree stacking [22,23] and the stacking model with weak learner stacking. Machine learning algorithms such as long short-term memory networks and other deep learning algorithms are used to build a multi-model fusion ensemble learning model [24]. In terms of power system probabilistic prediction, good prediction results have also been achieved by combining an integrated learning model with a quantile regression model to achieve nonlinear system wind power output prediction.

In summary, in view of the multi-value quantile prediction crossover problem and the multi-objective prediction performance degradation problem, a load probabilistic prediction method based on a combination of multi-value quantile regression and an integrated learning model is proposed. First, a time series fusion ensemble learning model is constructed to extract complex timing features in a 10 kV bus load; secondly, multi-value quantile regression is used as the objective function to obtain multiple-quantile predicted values for future time points of the 10 kV bus load; finally, a maximum likelihood sorting constraint is introduced based on the multi-value quantile regression loss function to optimize quantile sorting, and kernel density estimation is used to obtain the continuous probability density value and cumulative distribution curve of the multi-value quantile. Probabilistic prediction is performed on the 10 kV bus load of a distribution network. The results show that, compared with commonly used load probabilistic prediction methods, this method can reduce the crossing of multi-value quantiles, improve the performance of the prediction model, and ultimately improve the reliability and accuracy of the probabilistic prediction results.

The contributions of this paper are summarized as follows: (1) We propose an MQR-ELM framework. This framework innovatively adopts a stacking ensemble architecture, using multiple TFN sub-models to extract, in parallel, the multi-dimensional time-series features of a load. This significantly enhances our ability to extract the dynamic features of a load. At the same time, predicting multiple-quantile values can comprehensively depict the probability distribution of a load, effectively improving computational efficiency. (2) We embedded the ListMLE constraint into the loss function of the MQR. By imposing a monotonicity constraint during the model training phase, we effectively mitigated the quantile crossing problem in multi-quantile prediction. (3) We combined the temporal fusion integration framework with MQR embedded with the ListMLE constraint and verified it using one-year electricity consumption data from a 10 kV line in Nanjing, Jiangsu Province. This effectively demonstrates the effectiveness and reliability of the model in load probability prediction.

To more clearly illustrate the positioning and advantages of the method proposed in this paper in the field of probabilistic load forecasting, Table 1 compares the method of this paper with representative methods in recent relevant studies. Existing non-parametric probabilistic forecasting models (Refs. [16,17]) have limited capabilities in extracting complex time-series features and are highly prone to the quantile crossing problem when dealing with multi-level quantile forecasting. Although some studies (Ref. [19]) attempt to solve the crossing problem by rearranging quantiles through post-processing, they do not address the quantile crossing problem at the model level. A few studies that introduce ensemble learning (Ref. [22]) focus on simple quantile superposition and lack in-depth parallel extraction of multi-dimensional time-series features. In contrast, the method of this paper uses ListMLE to constrain the objective function and utilizes an ensemble temporal fusion learning model to achieve the extraction of complex time-series features for multi-objective quantile forecasting.

## 2. Integrated Learning Model and Multi-Value Quantile Regression Principle

**Figure 1:**
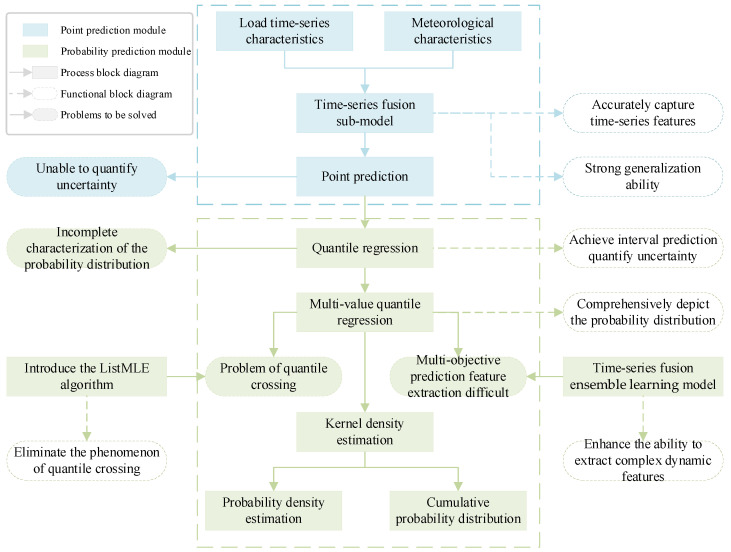
Conceptual roadmap of this paper.

In order to clearly clarify the logical progression and integration relationship of various technical components within the proposed framework, Figure 1 shows a conceptual roadmap of this paper.

Firstly, although traditional point prediction models have strong generalization abilities and the ability to capture time-series characteristics, they cannot quantify the uncertainty of predictions. To this end, quantile regression is introduced to achieve probabilistic prediction to quantify uncertainty. However, traditional quantile regression does not fully characterize the probability distribution. Therefore, multi-value quantile regression is adopted to comprehensively depict the probability distribution of the load. However, the application of multi-value quantile regression comes with two major challenges: First, the independent prediction of multiple quantiles is extremely likely to lead to the problem of quantile crossing. To mathematically eliminate this phenomenon, this paper introduces the ListMLE algorithm to constrain the monotonicity of quantiles. Second, multi-objective quantile prediction makes it difficult to extract load features. The temporal fusion integrated learning model can effectively extract the complex dynamic features of the load. Finally, kernel density estimation is used to transform the optimized discrete quantile output into a continuous probability density and cumulative probability distribution.

### 2.1. Multi-Value Quantile Regression

The foundation of multivariate quantile regression is traditional quantile regression. Unlike conventional least squares regression, traditional quantile regression estimates conditional distributions at different quantile levels. Through quantile regression, it is possible to analyze the relationship between dependent and independent variables across quantiles, as shown in Equation (1).

(1) $$y_{i}^{q}=F_{i}^{-1}(q|x_{i})$$

(2) $$F_{i}=P(y_{i}\leq y_{i}^{q}|x_{i})$$

where $y_{i}^{q}$ is the quantile value of the *i* load point in quantile *q*; $F_{i}$ is the cumulative distribution function of $y_{i}$ when the conditional input is $x_{i}$, that is, the probability corresponding to load $y_{i}$ when the value is less than the quantile value $y_{i}^{q}$; and $F_{i}^{-1}$ is the inverse function of the cumulative distribution function of probabilities.

Traditional quantile regression typically employs the Pinball Loss (PL) function in conjunction with neural network models, where PL serves as the neural network’s loss function (as shown in Equation (3)). This loss function is computationally tractable and demonstrates strong performance when modeling data with varied quantile levels. The neural network-based quantile regression models are established by optimizing network parameters through loss minimization during the training process.

(3) $$L_{q}(y_{i},f(x_{i}))=\left\{\begin{array}{c} (q-1)\cdot (y_{i}-f^{q}(x_{i})),y_{i}-f^{q}(x_{i})<0\\ q\cdot (y_{i}^{q}-f^{q}(x_{i})),\;\;y_{i}-f^{q}(x_{i})\geq 0 \end{array}\right.$$

where $L_{q}$ is the PL corresponding to quantile *q* when the true load value is $y_{i}$; $f^{q}(x)$ is the conditional quantile prediction model when the quantile value is *q*.

Traditional quantile regression can only construct conditional quantile regressions with a single quantile value, while multi-quantile regression models use multi-output models to simultaneously predict multiple quantile values of the load at a certain time:

(4) $$y_{i}^{q_{j}}=g(x_{i}),\;\;\;\;\;\;j=1,2,\ldots ,m$$

where $y_{i}^{q_{j}}$ is the load quantile value at the load moment *i* and the quantile value $q_{j}$; g(x) is a multi-value quantile prediction model that can simultaneously predict *m* quantile values of the load at time *i*; the corresponding multi-value quantile regression loss function is shown in Equation (5), which is the cumulative sum of the loss of multiple quantiles. It can be regarded as a multi-objective loss function of equal weight.

(5) $$ML(y_{i},f(x_{i}))=\sum_{j=1}^{m}L_{q_{j}}(y_{i},g(x_{i}|q_{j}))$$

To intuitively understand the output of the multi-value quantile regression model, it is essential to clarify the probabilistic meaning of the generated quantiles. At any given future instant, a predicted quantile corresponding to the probability level q (0<q<1) represents the value below which the actual load is expected to fall with a probability of q×100%. By simultaneously predicting a dense and uniformly spaced set of discrete quantiles (from q=0.01 to q=0.99), the model effectively constructs a discrete approximation of the full cumulative distribution function for the future load. Furthermore, prediction intervals at any specific confidence level can be directly derived from these quantile outputs. For example, a 95% prediction interval is obtained by extracting the bounds between the 0.975th quantile and the 0.025th quantile. This comprehensive output format provides a complete spectrum of load uncertainty.

### 2.2. Listwise Maximum Likelihood Estimation Sorting Algorithm

The objective function of multi-value quantile load prediction is that the load value of multiple quantiles should obey the characteristics of monotonicity, that is, the higher the quantile, the larger the predicted value. In interval prediction, the 95% confidence interval should fully contain the 90% confidence interval, but in the actual prediction process, due to data complexity and a lack of constraints on the objective function during the prediction process, its monotonicity may no longer be true. In interval prediction, the 95% confidence interval does not completely contain the 90% confidence interval. This phenomenon is called the quantile value crossing phenomenon. The maximum likelihood estimation sorting algorithm (Listwise Maximum Likelihood Estimation, ListMLE) is a machine learning method used for sorting tasks. It can formalize ranking problems as a function of maximizing the likelihood between a predicted list and a real list, implement optimization of the sorting of the list data, and maximize the probability of correct sorting, thus improving the sorting quality of the entire list [25].

In the multi-value quantile regression problem, for a time when the input feature is *X*, the predicted value vector of *m* quantiles of the load is denoted as *Y*, and *P_XY_* is the unknown but fixed joint probability distribution of *X* and *Y*. In this paper, the Plackett–Luce model is used to construct the probability distribution, which describes the order of the predicted load quantile values. The details are shown in Formula (6).

(6) $$P(y_{i}^{q_{j}}|x_{i};g)=\prod_{j=1}^{m}\frac{\exp(g(x_{y(i)}))}{\sum_{k=1}^{m}\exp(g(x_{y(k)}))}$$

The sorting constraint for multi-value quantiles can be converted to minimize the expected sorting loss *R*(*h*):

(7) $$R(h)=\int_{X\cdot Y}l(h(g(x_{i}|q_{j})),y_{i}^{q_{j}})dP(x_{i},y_{i})$$

where $P(x_{i},y_{i})$ is the joint probability density distribution; *h* is the multi-quantile value ordering function, whose definition can be referred to Equation (8); and *l* is the 0–1 loss function, whose definition is shown in Equation (9).

(8) $$h(x_{i}|q_{j})=s(g(x_{i}|q_{1}),g(x_{i}|q_{2}),\ldots ,g(x_{i}|q_{j}))$$

(9) $$l(h(x_{i}|q_{j}),y_{i}^{q_{j}})=\left\{\begin{array}{l} 1,\;h(x_{i}|q_{j})\neq y_{i}^{q_{j}}\\ 0,\;h(x_{i}|q_{j})=y_{i}^{q_{j}} \end{array}\right.$$

where *s* indicates that when the input is $x_{i}$, the predicted multiple load quantile values are reordered. Due to the fact that the integral operation of the 0–1 loss and sorting function $h(x_{i}|q_{j})$ in actual calculations will greatly increase the computational difficulty of the model and lead to optimization difficulties, this paper replaced the original sorting loss function by constructing a likelihood loss function *Φ* [26]. This function is also known as the negative logarithmic loss function, as shown in Equation (10). Based on the definition of the ranking loss function for the maximum likelihood estimation, as shown in Equation (11), the multi-value quantile values output by the model are sorted by maximizing the probability.

(10) $$\phi (g(x_{i}|q_{j}),y_{i}^{q_{j}})=-\log P(y_{i}^{q_{j}}|x_{i};g)$$

(11) $$R^{\phi }(g)=\frac{1}{m}\sum_{j=1}^{m}\phi (g(x_{i}|q_{j}),y_{i}^{q_{j}})$$

The multi-value quantile regression loss function is constructed, as shown in Equation (12), which includes two parts: multi-value quantile regression loss and ranking constraint loss.

(12) $$L(y_{i},f(x_{i}))=\sum_{j=1}^{m}L_{q_{j}}(y_{i},g(x_{i}|q_{j}))+R^{\phi }(g)$$

Through the above steps, ListMLE’s likelihood loss function adaptively enforces quantile ordering constraints during optimization, ensuring that higher quantile predictions consistently dominate their lower counterparts. This adaptive mechanism not only enhances model interpretability but also improves predictive accuracy. Furthermore, the convexity of ListMLE combined with its computational efficiency guarantees rapid convergence across large-scale datasets, while maintaining robust stability and reliability throughout the optimization process.

In summary, by applying ListMLE to the loss function of multi-value quantile regression, the quantile crossover problem can be effectively reduced, and the interpretation and prediction accuracy of the model can be improved. This method can not only deal with complex ranking problems but can also achieve efficient optimization on large-scale data sets and provide more reliable and accurate forecast results for bus load probabilistic prediction of power systems.

### 2.3. Timing Fusion Integrated Learning Model

**Figure 2:**
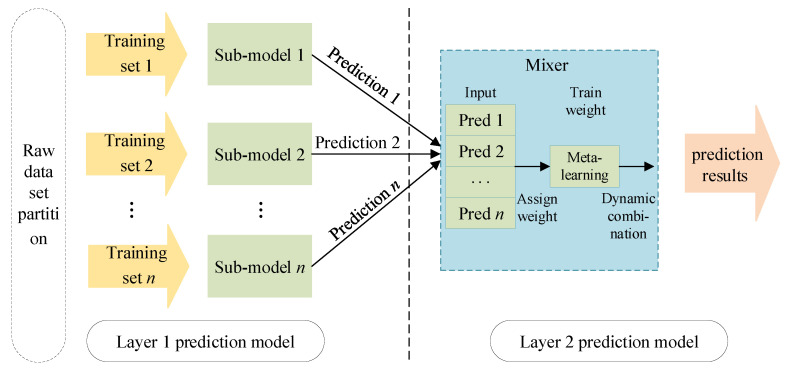
Ensemble learning model based on stacking.

The stacking-based integrated learning framework first divides the original dataset into several sub-datasets and inputs them into each sub-model of the layer 1 prediction model. Each sub-model will output its own prediction results. Then, the output of layer 1 is used as the input of layer 2 to train the mixer of layer 2 to obtain the final prediction result. The stacking framework can optimize the output results of multiple models to achieve the improvement of overall prediction accuracy. The model structure is shown in Figure 2.

#### 2.3.1. Timing Fusion Sub-Model

**Figure 3:**
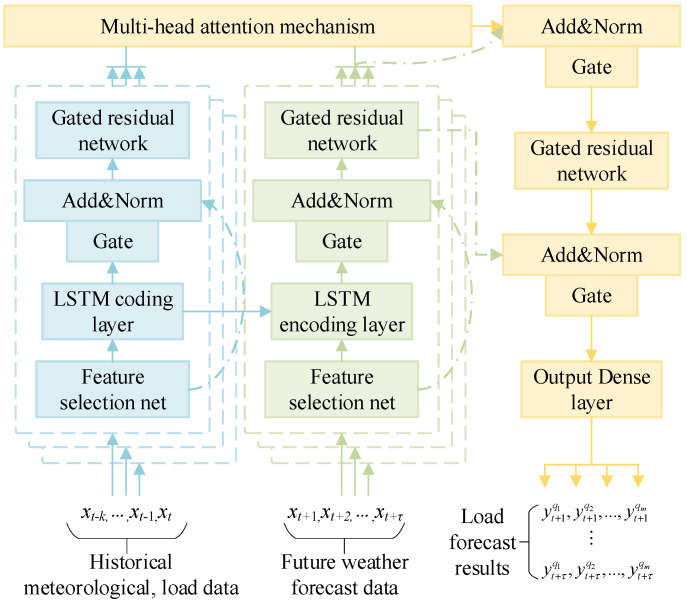
Temporal fusion sub-model network architecture.

The design of sub-models has a major impact on the performance of ensemble learning models. Too complex a model will lead to a decline in model generalization ability and a sharp increase in time costs, while too simple a model will lead to a decline in the predictive accuracy of the model. In this paper, the neutron model adopts a temporal fusion network (TFN) [27], this model can make full use of multi-dimensional temporal features and process both historical temporal feature vectors and future temporal feature vectors by constructing multiple inputs. The structure is shown in Figure 3, where the solid arrows indicate the model data transmission process and the dotted arrows indicate the residual connection structure.

Load prediction results for the learning model: *n* is the number of sub-models in the integrated learning model and is an adjustable hyperparameter of the model, but does not exceed the prediction number of the multi-value quantile *m*. The sub-model contains two parts of the input. The first part of the input is a known historical feature vector, which contains past meteorological factors, load levels, holiday information, etc.; the other part is a feature vector corresponding to the load time to be predicted, that is, the predicted values of future meteorological conditions, holiday information, etc. Each sub-model is trained through the first input part, extracting meteorological characteristics and load multi-dimensional timing characteristics, and the training process of each sub-model is independent of the others. Secondly, the future load is predicted through the second input part; the multi-value quantile regression loss function shown in Formula (12) is used as the loss function of the sub-model, and each sub-model can obtain the respective multi-value quantile regression prediction result.

After an input historical timing feature vector is selected through the feature network, the LSTM encoding layer is used as part of the decoding process. This process uses the short-term change features of the load as part of the load prediction decoding layer, and then uses a gated residual network to perform feature extraction of the coded vector. After using the same operation as the one used for the future feature vector, the long-term change information of the load is extracted from the multi-head attention mechanism layer. Short-term change information is extracted, and the short-term change information is integrated with the long-term load change information, which together constitute the fusion of the long-term and short-term timing features in the sub-model.

**Figure 4:**
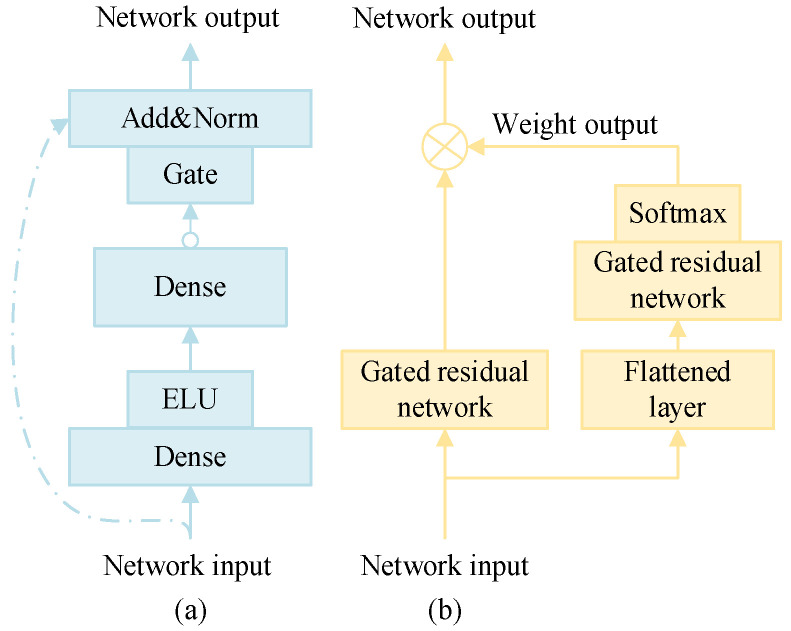
Gated residual network and feature selection network structure. (**a**) Gated residual network. (**b**) Feature selection network.

1. Gated residual network:

In the load prediction model, the characteristics of the load and the multi-value quantile prediction value are dynamically correlated; there are complex variable relationships, and it is difficult to determine the degree of nonlinear processing. The introduction of the gated residual network (GRN) can improve the flexibility and adaptability of the model. The structure is shown in Figure 4a.

The GRN introduces a nonlinear processing mechanism that can adaptively determine when to incorporate nonlinearity based on the characteristics and requirements of the data. This adaptive capability helps prevent the construction of overly complex models, particularly when dealing with small or noisy datasets, thereby reducing the risk of overfitting. The output of the GRN is given by Equation (14).

(13) $$r_{1}=\mathrm{ELU}(W_{1}x_{1}+b_{1})$$

(14) $$\mathrm{GRN}(x_{1})=\mathrm{LN}(x_{1}+G(W_{2}r_{1}+b_{2}))$$

where ***x***_1_ is the input feature vector of the network; **W**_1_, ***b***_1_, **W**_2_, and ***b***_2_ are the weight matrix and bias term vectors of the fully connected layers Dense 1 and Dense 2; ELU represents the ELU activation function, which is calculated as shown in Equation (15); LN is a normalized calculation; and G is the gated unit calculation, which can control the nonlinear transformation process of features. The calculation formula is shown in Equation (16).

(15) $$\mathrm{ELU}(x)=\left\{\begin{array}{cc} e^{x}-1 & x<0\\ x & x\geq 0 \end{array}\right.$$

(16) $$G(x)=\mathrm{Sigmoid}(W_{a}x+b_{a})⊙(W_{b}x+b_{b})$$

where Sigmoid is the activation function, ***x*** is the input feature vector; **W**_a_, ***b***_a_, **W**_b_, and ***b***_b_ are the weight matrix and bias term vectors of Dense a and Dense b in the fully connected C-layer, respectively; and ⊙ is the Hadamard product of the vectors.

2. Feature selection network

The feature selection network imposes constraints on the model’s input features, aiming to automatically identify and select the most relevant features for load forecasting during the training process. This mechanism reduces redundant information and enhances the model’s generalization capabilities. Compared to a standard fully connected layer, GRN improves feature selection by incorporating gating mechanisms that autonomously determine whether a given feature should be input into the network. This selective process mitigates the impact of irrelevant or redundant features, thereby enhancing generalization performance and reducing computational costs. This structure is illustrated in Figure 4b.

The input feature vector is first processed through a flattening layer and GRN, which outputs a weight for each feature, as defined in Equation (17). Subsequently, the feature vector—after undergoing nonlinear transformation via the GRN—is subjected to a weighted summation operation. This process enables the selection of input features for load forecasting, as expressed in Equation (18).

(17) $$\omega_{x_{t}}=\mathrm{Softmax}(G(\mathrm{Flatten}(x_{t})))$$

(18) $${\tilde{x}}_{t}=\sum \omega_{x_{t}}⊙G(x_{t})$$

where ***x****_t_* is the feature input vector at time *t*; $\omega_{x_{t}}$ is the weight and weight vector of the output, whose dimension is consistent with that of the feature vector after nonlinear processing at time *t*; and ${\tilde{x}}_{t}$ is the feature vector of the input load forecasting sub-model after feature selection.

#### 2.3.2. Ensemble Learning Mixer

In an integrated learning model, the function of the mixer is to synthesize the prediction results of multiple timing fusion sub-models to obtain the optimal prediction results. This article integrates a learning mixer to adopt a dynamic weighted structure, and the weights of each sub-model are continuously adjusted as trainable parameters during the model training process, ultimately improving the model’s prediction accuracy for multiple-quantile prediction targets. The prediction result of defining the *k*-th sub-model is $y_{k}$, as shown in Equation (19), and the mixer weighting calculation process is shown in Equation (20).

(19) $$y_{k|i}={\left[y_{i}^{q_{1}},y_{i}^{q_{2}},\ldots ,y_{i}^{q_{j}}\right]}_{K}^{T}$$

(20) $$y_{i}=\frac{1}{N}\sum_{k=1}^{n}\omega_{k}y_{k};\;\sum_{k=1}^{n}\omega_{k}=1$$

where $y_{i}^{q_{j}}$ is the predicted value of quantile $q_{j}$ at moment *I*; $y_{k}$ is the predicted value of the quantile load of the *k*-th model; $\omega_{k}$ is the weight of the prediction result of the *k*-th model; and $y_{i}$ is the output result of the mixer.

## 3. Load Probability Forecasting Model

**Figure 5:**
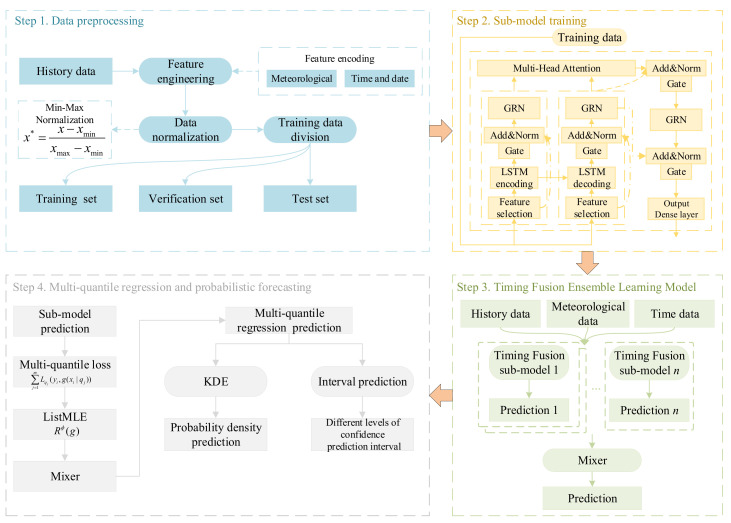
Load probabilistic prediction model.

The load probabilistic prediction model based on the stacking ensemble learning model is shown in Figure 5.

The probabilistic prediction model consists of three parts, data preprocessing, an integrated learning model, and load probabilistic prediction, and finally obtains the load probability density and load accumulation probability distribution.

Data preprocessing, as the first part of the load probability model, is a basic step in the training and verification of the model. In the model, time and date characteristics, meteorological characteristics and historical load characteristics were constructed as inputs to the model based on the load change characteristics, and min–max normalization was used for data preprocessing. The formula is as follows:

(21) $$x^{*}=\frac{x-x_{\min}}{x_{\max}-x_{\min}}$$

where $x^{*}$ is the normalized data; *x* is the original data; and $x_{\max}$ and $x_{\min}$ are the maximum and minimum values in the original data.

The specific input characteristics are shown in Table 2. To avoid the model from up-regulating parameters in the test set, before model training, the data of the test set was divided separately into a comparison reference for model performance, and the remaining data was re-divided into a training set and verification set. The model parameters were updated through the training set data, and the verification set was used to adjust the hyperparameters of the model.

The second part of the load probabilistic prediction model is the sub-model training process. In this phase, individual sub-models were trained independently, and the output result of each sub-model was the preliminary multi-value quantile prediction of the load.

The third part is the timing fusion ensemble learning model phase, which constitutes the mixer training process. This step functions by dynamically weighting the discrete prediction results generated by the multiple sub-models in the previous step. By actively optimizing the weight of each sub-model, the mixer generated an integrated prediction. Through this sequential ensemble learning structure, we finally obtained optimized and highly reliable multiple-quantile prediction results for a load at a given future moment.

It is worth noting that although all sub-models share an identical macroscopic TFN architecture, sufficient ensemble diversity is strictly guaranteed by several underlying mechanisms. First, the highly non-convex nature of the multi-value quantile regression loss landscape, combined with the random initialization of neural network weights for each sub-model, ensures that individual models converge to distinctly diverse local optima. Second, varying data partitions during the training phase expose each sub-model to different data distributions. Finally, the dynamic weighting mixer acts as an adaptive heterogeneous integrator, exploiting these diverse feature representations to effectively mitigate the predictive variance of any single sub-model.

The fourth part of the model was to convert the discrete quantile prediction value into a continuous probability density curve through the kernel density estimation method [28]. The kernel density estimation calculation is shown in Equation (22), and then the continuous probability distribution curve was obtained through the approximate integral algorithm as the prediction result of the load probabilistic prediction model.

(22) $${\hat{P}}_{h}(y_{i})=\frac{1}{m}\sum_{j=1}^{m}K_{h}(y_{i}-y_{i}^{q_{j}})$$

where *m* is the number of multi-value quantile predictions of the model; $y_{i}$ is the actual load value at the *i* time; $y_{i}^{q_{j}}$ is the quantile predicted value of the corresponding load quantile; and $K_{h}$ is the kernel function corresponding to the kernel density estimation, whose definition is shown in Equation (23).

(23) $$K_{h}(x)=\frac{1}{\sqrt{2\pi }}\exp(-x^{2}/2h^{2})$$

where the width is *h*; *x* is the sample value.

## 4. Example Analysis

### 4.1. Prediction and Evaluation Index

The evaluation indicators of the prediction results are shown in Table 3.

In this paper, PICP is used to describe the ratio of the true value that overlaps with the upper and lower bounds of the prediction interval with a given confidence interval. The difference between the PICP and the nominal confidence interval is the ACE [29], which is calculated by the formula shown in Equations (24) and (25).

(24) $$PICP(1-\alpha )=\frac{1}{n}\sum_{t=1}^{n}c_{1-\alpha }(y_{t})$$

(25) ACE(1−α)=(1−α−PICP(1−α))×100%

where $c_{1-\alpha }$ indicates that when the confidence is *α*, it is judged whether the true value of load $y_{t}$ at time *t* is within the confidence interval (1 − *α*). The answer “Yes” corresponds to 1, and “no” is 0. ACE is the deviation between the forecast confidence interval and the nominal confidence interval, and the smaller it is, the more accurate the prediction interval is.

In this paper, PIAW was used to describe the mean width of the prediction result of the confidence interval, and the calculation formula is shown in Equation (26).

(26) $$PIAW(1-\alpha )=\frac{1}{n}\sum_{t=1}^{n}\left(U_{t}^{1-\alpha }-L_{t}^{1-\alpha }\right)$$

where $U_{t}^{1-\alpha }$ and $L_{t}^{1-\alpha }$ are the upper and lower bounds of the prediction interval when the confidence interval is *α*, respectively. This index is used to measure the prediction width of the model under a certain confidence interval. The smaller the value, the smaller the prediction width of the model.

Since interval coverage and average width are two different optimization objectives, the Winkler Score [30] is used as the comprehensive evaluation index of interval prediction, and the two prediction performance of interval coverage and average prediction width of the comprehensive evaluation model are evaluated.

(27) $$WS=\frac{1}{n}\sum_{t=1}^{n}S_{t}^{1-\alpha }$$

where $S_{t}^{1-\alpha }$ is the interval prediction fraction corresponding to time *t* when the confidence is *α*, and its calculation formula is shown in Equation (28). The smaller the value, the higher the coverage rate and the smaller the prediction width of the model under the same confidence interval.

(28) $$S_{t}^{1-\alpha }=\left\{\begin{array}{l} (U_{t}^{1-\alpha }-L_{t}^{1-\alpha })+2(L_{t}^{1-\alpha }-y_{t})/\alpha ,\;y_{t}<L_{t}^{1-\alpha }\\ U_{t}^{1-\alpha }-L_{t}^{1-\alpha },\;\;\;\;\;\;\;L_{t}^{1-\alpha }\leq y_{t}\leq U_{t}^{1-\alpha }\\ (U_{t}^{1-\alpha }-L_{t}^{1-\alpha })+2(y_{t}-U_{t}^{1-\alpha })/\alpha ,\;y_{t}>L_{t}^{1-\alpha } \end{array}\right.$$

CRPS [31] is adopted as the overall evaluation index of probabilistic prediction results for each model, as shown in Equation (29).

(29) $$CRPS(h)=(\frac{1}{nM}\sum_{t=1}^{n}\sum_{i=1}^{M}\left|y_{t+h}^{\left(i\right)}-y_{t+h}\right|)-(\frac{1}{2nM}\sum_{t=1}^{n}\sum_{i=1}^{M}\left|y_{t+h}^{\left(i\right)}-y_{t+h}^{(i)\prime }\right|)$$

where $y_{t+h}^{\left(i\right)}$, $y_{t+h}^{(i)\prime }$ are the sample values of the distribution ${\hat{F}}_{t,h}^{-1}(\tau )$; $y_{t+h}$ is the true sample value; *M* is the number of samples; and *n* is the forecast time step. The smaller the CRPS value, the better the sharpness of the load probability forecast result. That is, the probabilistic prediction results are more concentrated near the real load value.

CDI [32] is used to describe the degree of crossing between quantile values [33], which is used to evaluate the reliability of probabilistic prediction quantile values. Its calculation formula is shown in Equation (30).

(30) $$CDI=\frac{100}{\left|n\right|}\sum_{\begin{array}{c} q_{w},q_{v}\in Q\\ q_{v}<q_{w} \end{array}}\sum_{i}^{n}(q_{w}-q_{v}),\;y_{i}^{q_{w}}<y_{i}^{q_{v}}$$

where $q_{w}-q_{v}$ indicates the degree of deviation of quantiles between two crossing quantiles. The smaller the value, the smaller the degree of crossover between the two crossing quantiles, and the value is 0 when there is no crossing.

### 4.2. Experimental Data Set and Platform

**Figure 6:**
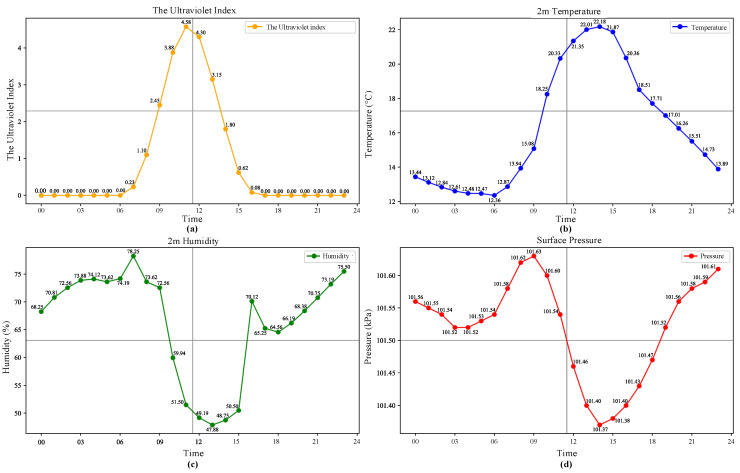
Sample meteorological data. (**a**) Daily ultraviolet index data. (**b**) Daily temperature data. (**c**) Daily humidity data. (**d**) Daily surface pressure data.

The experimental dataset in this paper uses load electricity data from a 10 kV line in Nanjing City, Jiangsu Province. The load data was acquired from 1 November 2017 to 31 October 2018, with a collection interval of 15 min. The corresponding meteorological data comes from the NASA Surface Meteorology and Solar Energy Dataset, including ultraviolet radiation exposure index, temperature, humidity and air pressure, etc., with a collection interval of 15 min. The models used in this article are all built on the Keras and Tensorflow deep learning platforms in Python 3.11.7. Figure 6 shows the meteorological data of a sample day.

### 4.3. Model Training and Parameter Setting

To verify the performance of this model, the comparison model uses the parameter model Mixture Density Network (MDN) model, the non-parametric Quantile Regression Neural Network (QRNN) model, and the improved algorithm quantile regression gated recurrent unit model (QRGRU). In order to verify the improvement effect of the sorting algorithm and ensemble model on multi-value quantile regression prediction, the comparison model type and parameter settings were set as shown in Table 4. The last month’s data was used as test data, including holidays, weekdays and weekends, and the rest of the data was used as training verification data, with the ratio of training data and verification data being 7:3. Each model predicted load quantile values of 0.01~0.99 under a single time step in advance and further used kernel density estimation to estimate the probability density of load changes.

The model optimization algorithm adopts the Adam algorithm. In order to make the model reach the optimal state during training, the total number of iterations of the model in this paper was set to 100, and an automatic termination algorithm was used; when the model’s training loss function no longer showed a downward trend in multiple consecutive rounds, the training was terminated.

The initial learning rate of the model was set to 0.001, and an exponential function attenuation scheduling mode was used to monitor for a situation where the loss function did not decrease during the training process. When the loss function no longer decreased, the learning rate was automatically reduced so that the model continued to be optimized at a lower learning rate, ensuring that the model could fully converge and obtain the best performance.

### 4.4. Load Probability Forecast Results

1. Comparison of interval prediction performance

**Figure 7:**
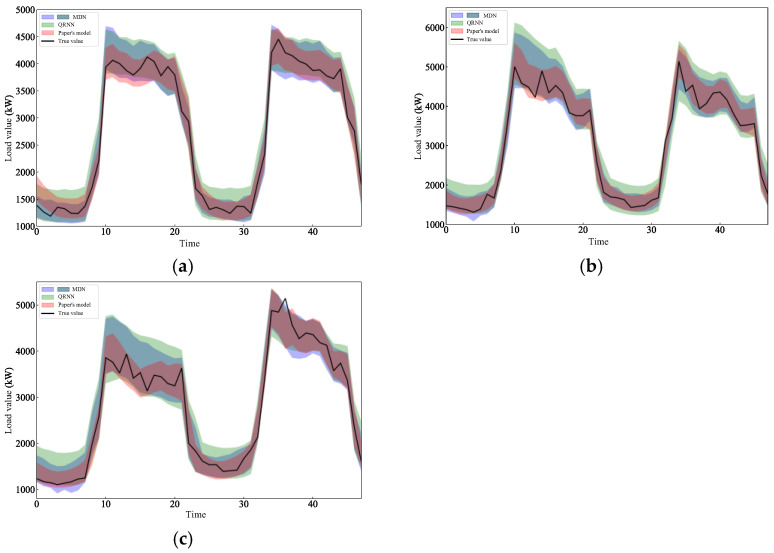
Prediction intervals of various models: (**a**) Forecast interval for 1 and 2 October. (**b**) Forecast interval for 9 and 10 October. (**c**) Forecast interval for 20 and 21 October.

Since there are many comparison models in this article, Figure 7 shows the prediction results of the 0.95th quantile regression of the test set by paper’s model and some other models, including the prediction results of three types of days: holidays on 1 and 2 October, working days on 9 and 10 October, and weekends on 20 and 21 October. Appendix A shows the prediction results of this model and the remaining models.

Comparing Figure 7 and Appendix A, it can be seen that the fluctuation range of holiday loads is smaller than for the two days before and after, making the model prediction width relatively narrower. For workday loads and weekend loads, due to the impact of large fluctuations at peak moments, the prediction interval width of the model becomes larger, but the prediction interval of this model, which is under the 0.95^th^ quantile, can basically cover the real load value, and the prediction interval width is more reasonable than that of other models.

A comparison of each model’s predicted 95% confidence interval and overall prediction performance results is shown in Table 5. It can be seen that the model in this paper effectively reduces the prediction interval width by introducing an ensemble model, and the coverage rate of the prediction interval (94.624%) is closer to the nominal coverage rate (95%), that is, the prediction interval is more accurate. Referring to the comprehensive evaluation index, the Winkler Score, we can also find that the use of an integrated model can improve the interval prediction performance of the model. Comparing the models QRR and QRL, compared with the introduction of common Rank constraints, the ListMLE constraint introduced in this paper plays a better role in constraining the prediction interval width of the model but leads to an increase in the deviation value. Comparing the QRN, QRNJ, TFL, and the model in this paper, it can be seen that the integrated learning model can effectively solve the problem of larger coverage deviations caused by the ListMLE algorithm and improve the comprehensive interval prediction performance.

2. Comparison of probabilistic prediction performance

**Figure 8:**
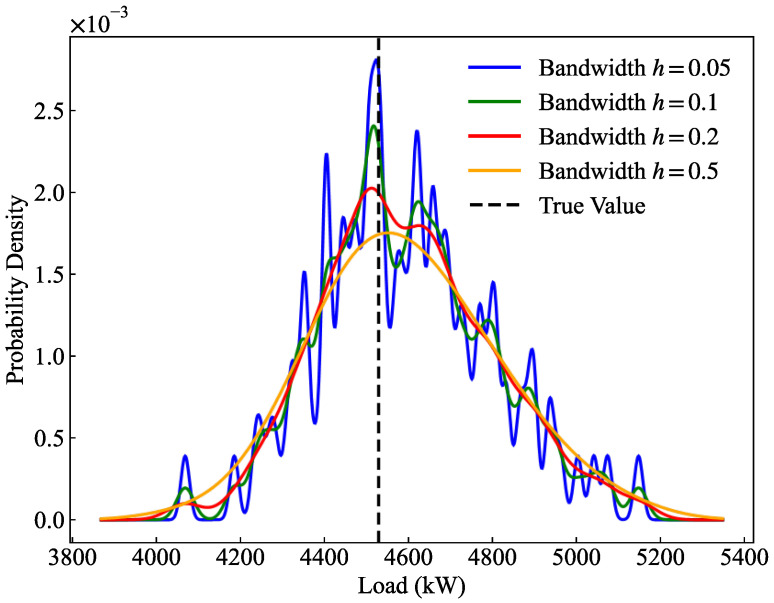
Sensitivity analysis of KDE bandwidth.

To evaluate the impact of the bandwidth parameter h in kernel density estimation on the estimated distributions and CRPS, a sensitivity analysis was conducted, as illustrated in Figure 8 and Table 6. Regarding the distribution shape, when h is excessively small (h=0.05, h=0.1), the probability density curve exhibits severe oscillations and multiple local spikes. This phenomenon indicates overfitting to the discrete quantile predictions, which violates the continuous and smooth physical nature expected of actual bus load probability distributions. Conversely, when h is excessively large (h=0.5), the curve demonstrates “over-smoothing,” significantly lowering the peak and unreasonably widening the distribution, resulting in a substantially degraded CRPS of 59.081.

Quantitatively, although the CRPS values for h=0.05 (52.81) and h=0.1 (53.14) are marginally lower than that of h=0.2 (54.06), this slight numerical gain comes at the unacceptable cost of sacrificing the physical rationality and generalization ability of the density curve. Considering the optimal trade-off between probabilistic sharpness, the physical smoothness of the distribution, and the ability to cover true values, setting the bandwidth to h=0.2 effectively eliminates local noise while preventing severe accuracy loss caused by over-smoothing. Therefore, h=0.2 is adopted as the optimal KDE bandwidth parameter in this study.

**Figure 9:**
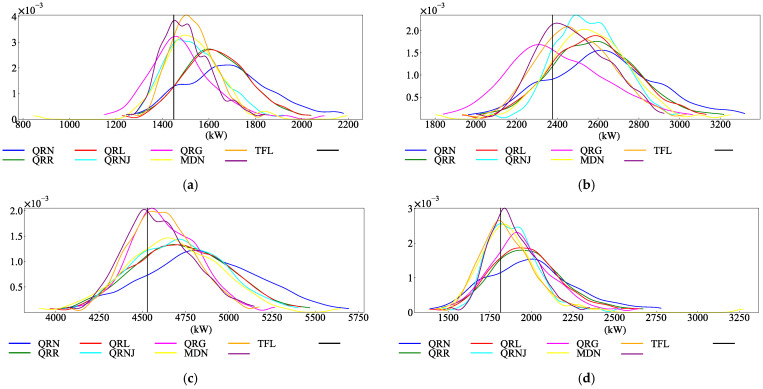
Probability density prediction results of each model: (**a**) Estimate of load probability density at 0 on 10.8. (**b**) Estimate of load probability density at 8 on 10.8. (**c**) Estimate of load probability density at 16 on 10.8. (**d**) Estimate of load probability density at 23 on 10.8.

Each model was used to predict the corresponding load quantile values under 0.01 to 0.99 quantiles at three moments on 8 October, and then the kernel density estimation calculation method was used to obtain the probability density prediction results, as shown in Figure 9. It can be seen from the figure that the probability density value of the proposed model is large at the true load value, and the estimated probability density value is close to the true value and relatively concentrated, which verifies that the proposed model can improve the reliability and accuracy of probabilistic prediction. By comparing the load probability density estimation at different time points, it can be found that the peak value of the probability density predicted by the model with poor performance is higher than the actual load value, and the predicted value tends to be higher than the actual value, showing a long tail phenomenon on the probability density curve. However, the model in this paper can describe load fluctuations more accurately.

In order to verify the effectiveness of the sorting algorithm for maximum likelihood estimation, CDI evaluation indicators among different models were compared, and the results are shown in Table 7. Since MDN is a parametric model, the quantile values generated by MDN were arranged from small to large, and the crossover phenomenon of quantile values did not need to be considered. It can be found that the index of the quantile crossing degree in the ratio table is the smallest. By further comparing the QRR model with the QRL model, it can be seen that the ListMLE quantile loss function constructed in this paper based on multi-value quantile regression was superior to directly introducing the Rank constraint of the general ranking algorithm. The results of the comparison with the QRNJ model and the model in this paper also show that the introduction of ensemble learning can effectively reduce the crossover degree of the model in multi-value quantile regression. Comparing the degree of crossover under different day types, load fluctuations on holidays were small, and the gap between the predicted quantile values was small, resulting in a large degree of crossover of the load quantile values of each model on holidays, while the situation on work days and weekends was the opposite to that of holidays, and the two were very similar.

The CRPS index was used to further compare the overall probabilistic prediction performance between different models on holidays. The smaller the value, the more reasonable the prediction result of the multi-value quantile model, as shown in Table 8. From the indexes of the QRNJ model and TFL model, we can see the advantages of the sorting constraint of maximum likelihood estimation and the intertemporal sequence fusion model. Moreover, by constructing the integrated learning model, the paper’s model achieves the optimal prediction result, which is slightly worse than the parametric MDN model (74.902) only for the prediction of holiday loads (75.303). However, the proposed model is obviously superior to the MDN model for load forecasting for other day types.

To verify the effectiveness and irreplaceability of the core parts of the method proposed in this paper, a targeted ablation experiment is designed. The verification is mainly carried out from three aspects: (1) Models with different ranking constraints are designed to independently verify the role of the ListMLE constraint in reducing the quantile crossing phenomenon. (2) Models with TFN time-series feature extraction are designed to verify the role of the TFN structure in capturing the complex multi-dimensional time-series features of bus loads and improving the probabilistic prediction performance. (3) Models with ensemble learning are designed to verify the role of the dynamic weighted ensemble strategy in achieving multi-objective prediction and improving the CRPS. The specific quantitative indicators and performance comparisons of the ablation experiment are shown in Table 9.

The results of the ablation experiment indicate that each core module in the MQR-ELM framework proposed in this paper plays an irreplaceable role in enhancing the model’s performance. Among them, the ListMLE constraint (QRL compared with QRN) significantly reduces the CDI, effectively solving the quantile crossing problem from a mathematical mechanism perspective. The TFN structure (TFL compared with QRL) significantly decreases the CRPS and WS, demonstrating its remarkable advantages in extracting complex multi-dimensional time-series features. The stacking ensemble learning architecture (the model in this paper compared with TFL) further surpasses the prediction variance of a single network, reducing all error indicators to the lowest level (CDI drops to 0.0476, and CRPS drops to 84.931).

**Figure 10:**
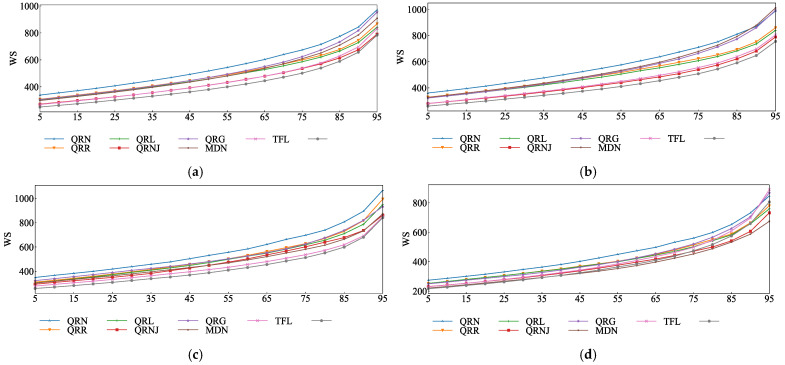
Probability density prediction results of each model: (**a**) Entirety of forecast. (**b**) Work day forecast. (**c**) Weekend forecast. (**d**) Holiday and festival forecast.

The probabilistic prediction performance of different models was evaluated using the Winkler Score under multi-value quantiles, as shown in Figure 10. The lower the curve’s values, the better the prediction performance of the model. It can be seen that the index curves of the prediction results of the model in this paper are all below those of other models for the different day types.

As shown in Figure 10a, the overall prediction results of the QRNJ model and TFL model are very close to each other in terms of their prediction performance, as well as the index curve of the model in this paper, which verifies the positive role of the ensemble learning model and the effectiveness of the time series fusion model. The Winkler Score index curves of various holiday load models, with relatively gentle changes, are also very close, indicating that the model in this paper has good forecasting capabilities when dealing with the probabilistic prediction of work day and weekend loads with large fluctuations.

## 5. Conclusions

Load probabilistic prediction is an important means to describe the load uncertainty of power systems. In this paper, a time-sequence fusion ensemble learning model with improved multi-value quantile regression is constructed, and the following conclusions are drawn by comparison of numerical examples:

1. Multi-value quantile regression based on the ranking algorithm of maximum likelihood estimation significantly reduces the crossover degree of multi-value quantiles, and the crossover index decreases by 53.786% compared with a traditional quantile regression model.
2. The temporal fusion ensemble learning method constructed in this paper solves the multi-objective problem corresponding to multi-value quantile regression well, reduces the interval width of the probabilistic prediction, and obtains more accurate coverage intervals.
3. By comparing examples of probabilistic predictions for loads on different types of days, the model in this paper reduces the deviation of the coverage rate of the probabilistic prediction interval and has a more significant improvement effect on probabilistic predictions for working days and weekends with large fluctuations.

Although the proposed MQR-ELM framework has achieved excellent forecasting performance on the 10 kV bus load dataset in Nanjing, this study still has certain limitations. Given that the experimental validation was based on a one-year dataset from a single geographic location and a single voltage level, the model’s performance may exhibit sensitivity to data heterogeneity and distributional shifts when applied to broader operating environments. For example, in extreme climatic zones (such as severely cold or tropical regions) or grid configurations with highly industrialized characteristics, the fluctuation patterns and meteorological sensitivities of the loads will change significantly. Nevertheless, the variable selection network built into the TFN within our framework can, to some extent, adaptively assign dynamic weights to different meteorological and temporal features, thereby providing a baseline of robustness for cross-regional applications. Future work will focus on compiling multi-source heterogeneous datasets encompassing diverse climatic zones, varying voltage levels (e.g., 35 kV or 110 kV), and different grid configurations to further validate and fine-tune the framework’s generalizability, as well as exploring cross-regional probabilistic load forecasting methods based on transfer learning.

## Figures and Tables

**Table 1:** Comparison between the paper’s methods and representative probabilistic forecasting models in the recent literature.

Recent Research	Method	Capability of Time-Series Feature Extraction	Quantile Crossing Method	Ensemble Learning Application
Refs. [16,17]	Quantile regression	Weak	-	-
Ref. [19]	Quantile regression forest	Mediocre	Post-processing reordering	-
Ref. [22]	Ensemble non-crossing quantile regression	Relatively strong	Loss function penalty adjustment	Simple fusion
Paper’s method	MQR-ELM	Strong	ListMLE	Stacking

**Table 2:** Feature input for probabilistic prediction model.

Feature Type	Paraphrase
Time date feature	It contains information about the month, day, hour, minute, and holiday corresponding to the load prediction points
Meteorological characteristics	It includes atmospheric temperature, relative humidity, human comfort index, and temperature and humidity indexes as meteorological factors
Historical load characteristics	The load value at multiple time points prior to the predicted load point

**Table 3:** Evaluation index of probabilistic prediction model.

Evaluation Indicators	Abbreviation	Paraphrase
Prediction Interval Coverage Probability	PICP	The proportion of the true value falling within the confidence interval
Average Coverage Error	ACE	Percentage deviation between PICP and theoretical expected coverage
Prediction Interval Averaged Width	PIAW	Average width of confidence interval of prediction results
Winkler Score	WS	Consider comprehensive indicators of PICP and PIAW
Continuous Ranked Probability Score	CRPS	Overall accuracy indicators by comparing the difference between predicted and true distributions
Crossing Degree Index	CDI	The degree of intersection between quantile values

**Table 4:** Load probabilistic prediction model parameter configuration.

Model	Abbreviation	Neural Network Layer	Integration Number
QRNN	QRN	Dense (100/200/500/500/200), Batch normalization	-
QRNN-Rank	QRR		-
QRNN-ListMLE	QRL		-
QRNN integration	QRNJ		5
QRGRU-ListMLE	QRG	Conv1D (32), GRU (128/128/64/32), Dropout (0.1) Dense (100/200/500/500/200), hybrid network number is 5	-
MDN	-		-
TFN-ListMLE	TFL	The number of layers in the TFN model is referred to in Figure 2, and the Dense layers in GRN are set to 128	-
Paper’s model	-		5

**Table 5:** Forecasting performance of various models for 95% load confidence intervals.

Model	PICP(%)	PIAW	ACE(%)	WS
QRN	97.446	885.039	2.446	971.859
QRR	96.237	746.770	1.237	867.820
QRL	96.371	724.779	1.371	843.137
QRNJ	95.027	611.240	0.427	790.848
QRG	95.968	807.549	0.968	953.775
MDN	95.699	717.223	0.699	908.729
TFL	94.355	624.938	−0.645	832.454
Paper’s model	94.624	588.526	−0.376	783.588

**Table 6:** Comparison of CRPS performance indicators under different bandwidths.

Bandwidth *h*	CRPS
0.05	52.81
0.1	53.14
0.2	54.06
0.5	59.08

**Table 7:** CDI of predicted quantile values of various models.

Model	CDI			
	Entirety	Working Day	Weekend	Holidays and Festivals
QRN	1.588	1.206	1.754	2.429
QRR	0.634	0.554	0.499	0.955
QRL	0.582	0.527	0.516	0.779
QRNJ	0.103	0.0834	0.0751	0.179
QRG	0.493	0.440	0.405	0.704
TFL	0.438	0.316	0.480	0.718
Paper’s model	0.0476	0.0332	0.0528	0.0802

**Table 8:** CRPS performance metrics for probabilistic forecasts of various models.

Model	CRPS			
	Entirety	Working Day	Weekend	Holidays and Festivals
QRN	115.737	120.494	116.682	92.468
QRR	103.030	108.736	105.491	85.632
QRL	101.170	106.389	103.548	85.292
QRNJ	91.574	93.113	98.745	78.742
QRG	104.455	109.968	107.951	83.803
MDN	101.751	111.449	99.742	74.902
TFL	90.566	94.333	92.020	79.561
Paper’s model	84.931	86.999	86.281	75.303

**Table 9:** Forecasting performance comparison of the ablation study models.

Type	Model	WS	CRPS	CDI
Models with different ranking constraints	QRN(no ranking constraints)	971.859	115.737	1.588
	QRR (with Rank)	867.820	103.0304	0.634
	QRL (with ListMLE)	843.137	101.170	0.582
Models with time-series feature extraction	QRL (no TFN)	843.137	101.170	0.582
	TFL (with TFN)	832.454	90.566	0.438
Models with ensemble learning	TFL (no ensemble learning)	832.454	90.566	0.438
	Paper’s model	783.588	84.931	0.0476

**Figure A1:**
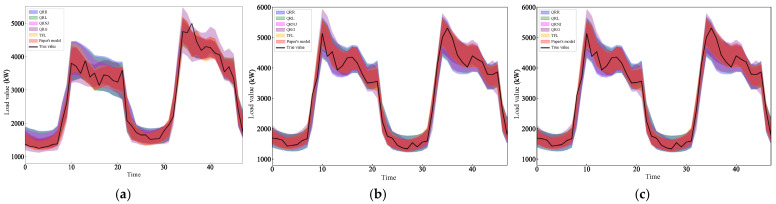
Prediction intervals of the paper’s model and other models: (**a**) Forecast interval for 1 and 2 October. (**b**) Forecast interval for 9 and 10 October. (**c**) Forecast interval for 20 and 21 October.

## Data Availability

The original contributions presented in this study are included in the article. Further inquiries can be directed to the authors.
